# Bortezomib-Induced Superficial Vasculitis in a Kidney Transplant Recipient: A Rare Case

**DOI:** 10.7759/cureus.58743

**Published:** 2024-04-22

**Authors:** Manish Balwani, Amit Pasari, Prasad Gurjar, Kapil Sejpal, Charulata Bawankule, Priyanka Tolani, Shubham Dubey, Pranjal Kashiv, Amol Bhawane, Sunny Malde, Sushrut Gupta

**Affiliations:** 1 Department of Nephrology, Saraswati Kidney Care Center, Nagpur, IND; 2 Department of Nephrology, Jawaharlal Nehru Medical College, Datta Meghe Institute of Higher Education & Research, Wardha, IND; 3 Department of Internal Medicine, Jawaharlal Nehru Medical College, Datta Meghe Institute of Higher Education & Research, Wardha, IND; 4 Department of Nephrology, All India Institute of Medical Sciences, Nagpur, Nagpur, IND

**Keywords:** immunosuppression, kidney transplant, antibody-mediated reaction, superficial vasculitis, bortezomib

## Abstract

Bortezomib is a frequently administered immunosuppressive agent in kidney transplantation. A 30-year-old male kidney transplant recipient developed an atypical reaction on the left hand in terms of spider-like extensions, indicating erythematous inflammation along the superficial veins after bortezomib intravenous administration. The inflammation spontaneously resolved after three weeks with a bortezomib dose reduction. Nephrologists must be familiar with the potential cutaneous bortezomib-induced adverse effects.

## Introduction

Drug-induced vasculitis may be limited to the skin but can also affect the lungs, kidneys, gastrointestinal tract, and peripheral nerves [1,2]. Multiple drugs can cause vasculitis, such as nonsteroidal anti-inflammatory drugs and antibiotics leading to isolated cutaneous leukocytoclastic vasculitis; hydralazine, cocaine, and antithyroid drugs causing antineutrophil cytoplasmic antibodies associated vasculitis; minocycline leading to medium-vessel vasculitis; anticancer drugs like immune checkpoint inhibitors causing large-vessel vasculitis; and sympathomimetic drugs leading to cerebral vasculitis [2]. It is challenging to distinguish drug-induced and unexplained vasculitis, but drug withdrawal is helpful to find out if vasculitis is drug-induced [1]. Here, we present a case of a 30-year-old male who developed vasculitis on the left or antibody-mediated rejection after one year of transplant after administration of bortezomib as a part of an anti-rejection regimen.

## Case presentation

**Figure 1:**
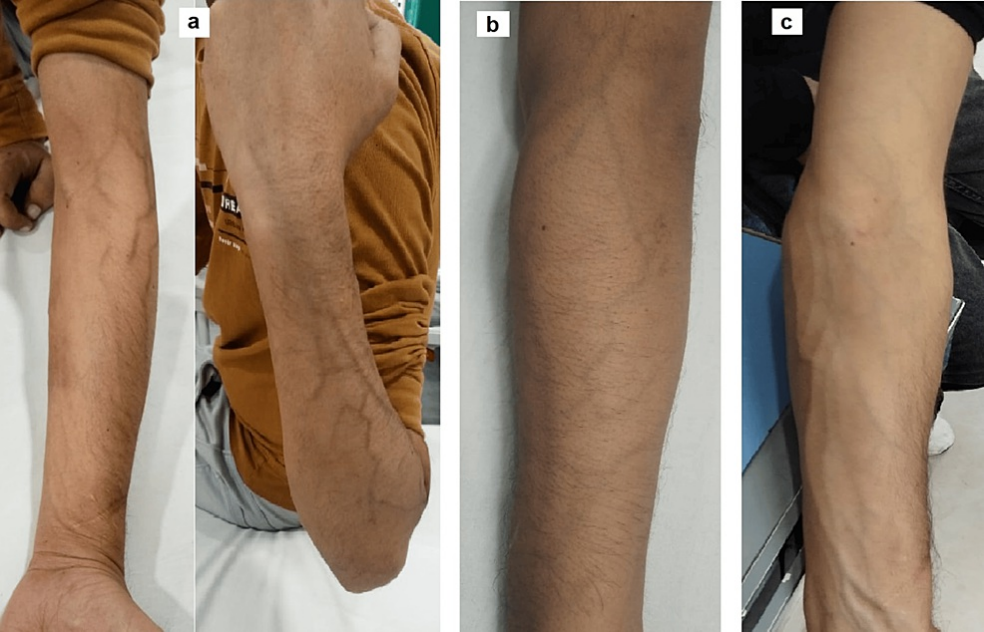
Bortezomib-induced cutaneous vein inflammation a: cutaneous vein acute inflammation manifesting as brownish spider web-like rash on ventral and dorsal surfaces of the upper limb; b: reduced intensity of inflammation with decreased rash intensity at one week; c: disappearing rash at two weeks.

A 30-year-old male who was on maintenance hemodialysis underwent a deceased donor transplantation and was started on standard maintenance immunosuppression. After a year of transplant, he developed acute antibody-mediated rejection (AMR). After immediate hospitalization, he received five sessions of plasma exchange and intravenous immunoglobulin. Subsequently, he was administered rituximab (500 mg) and anti-thymocyte globulin (50 mg), followed by bortezomib (2 mg) in four cycles. He developed an atypical reaction on his left upper limb during his fourth cycle of bortezomib without any associated severe symptoms, such as burning or itching. There were spiderlike extensions, indicating erythematous inflammation along the superficial veins. Given this, the dose of bortezomib was decreased to 3.5 mg/day, and the time of association of occurrence of this adverse event established the etiological role of bortezomib. Thus, we finally labeled the diagnosis as bortezomib-induced superficial vein inflammation. The dose of bortezomib was reduced. The skin inflammation was spontaneously resolved after three weeks (Figure 1). No new lesions were observed during the follow-up period.

## Discussion

Antibody-mediated rejection (AMR) after kidney transplantation is a significant complication that carries a poor prognosis and is most often a cause of post-transplant graft failure. The treatment strategy to counter AMR includes plasmapheresis, corticosteroids, and immunoglobulins, as well as defining a strategy for optimizing maintenance immunosuppression [3]. Bortezomib is a frequently administered immunosuppressive agent in kidney transplantation to reduce graft rejection. It is a proteasome inhibitor and acts by suppressing the T-cell immune response. It is commonly used in mantle cell lymphoma and multiple myeloma [4]. With bortezomib, adverse events ranging from skin rash to thrombocytopenia, leukopenia, and neutropenia are reported [5]. Bortezomib-induced skin issues, including cutaneous vasculitis, are common but poorly understood [6,7]. In bortezomib clinical trials, vasculitis and rashes are reported in 8-18% of patients. Skin vasculitis may not appear immediately. Some patients may experience rash in the second cycle and even in the seventh or ninth cycle of bortezomib [6].

The severe skin and systemic side effects caused by bortezomib are uncommon. Excessive secretion of inflammatory mediators, i.e., interleukin-6 (IL-6) and tumor necrosis factor-alpha (TNF-alpha), is possibly responsible for bortezomib-induced vasculitis. Administration of oral corticosteroids is effective in healing the vasculitis [6,7]. In our case, the patient was managed by reducing the dose of bortezomib. Dose optimization is crucial in the prevention of AMR [3]. There was a resolution of skin vasculitis within three weeks. Nephrologists must be familiar with such potential cutaneous bortezomib-induced adverse effects and understand that mere bortezomib discontinuation should not be the only preferred decision, as most cutaneous events might resolve spontaneously.

## Conclusions

The late occurrence of vasculitis after bortezomib administration is a rare adverse event. Such superficial skin vasculitis can resolve after a few weeks with dose optimization of bortezomib and ongoing immunosuppression. Counseling patients about the occurrence of such possible adverse events can be helpful in routine clinical practice.
